# COPD Clinical Control: predictors and long-term follow-up of the CHAIN cohort

**DOI:** 10.1186/s12931-021-01633-y

**Published:** 2021-02-04

**Authors:** Myriam Calle Rubio, Juan Luis Rodriguez Hermosa, Juan P. de Torres, José María Marín, Cristina Martínez-González, Antonia Fuster, Borja G. Cosío, Germán Peces-Barba, Ingrid Solanes, Nuria Feu-Collado, Jose Luis Lopez-Campos, Ciro Casanova, the CHAIN Study Investigators

**Affiliations:** 1 Pulmonology Department, Hospital Clínico San Carlos, C/ Martin Lagos S/N, 28040 Madrid, Spain; 2 Medical Department, School of Medicine, Universidad Complutense de Madrid, Madrid, Spain; 3 Respirology and Sleep Division, Queen’s University, Kingston, ON Canada; 4 Respiratory Department. Hospital, Universitario Miguel Servet and IISAragón, Ciber Enfermedades Respiratorias, Madrid, Spain; 5 Pulmonology Department, Hospital Universitario Central de Asturias, Universidad de Oviedo, Oviedo, Spain; 6 Pulmonology Department, Hospital Universitario Son Llàtzer, Palma de Mallorca, Spain; 7 Department of Respiratory Medicine, Hospital Universitario Son Espases-IdISBa and CIBERES, Palma de Mallorca, Spain; 8 Pulmonology Department, IIS-Fundación Jiménez Díaz-CIBERES, Madrid, Spain; 9 Pulmonology Department, Hospital de La Santa Creu Y San Pau, Universidad Autónoma de Barcelona, Barcelona, Spain; 10 Pulmonology Department, Hospital Universitario Reina Sofía, Instituto Maimónides de Investigación Biomédica de Córdoba, Universidad de Córdoba, Córdoba, Spain; 11 Unidad Médico-Quirúrgica de Enfermedades Respiratorias, Instituto de Biomedicina de Sevilla (IBIS), Hospital Universitario Virgen del Rocio, Universidad de Sevilla, CIBERES, Seville, Spain; 12 Pulmonology Department, Hospital Universitario Nuestra Señora de Candelaria, Universidad de La Laguna, Tenerife, Spain

**Keywords:** Chronic obstructive pulmonary disease, Control, Management

## Abstract

**Background:**

Control in COPD is a dynamic concept that can reflect changes in patients’ clinical status that may have prognostic implications, but there is no information about changes in control status and its long-term consequences.

**Methods:**

We classified 798 patients with COPD from the CHAIN cohort as controlled/uncontrolled at baseline and over 5 years. We describe the changes in control status in patients over long-term follow-up and analyze the factors that were associated with longitudinal control patterns and related survival using the Cox hazard analysis.

**Results:**

134 patients (16.8%) were considered persistently controlled, 248 (31.1%) persistently uncontrolled and 416 (52.1%) changed control status during follow-up. The variables significantly associated with persistent control were not requiring triple therapy at baseline and having a better quality of life. Annual changes in outcomes (health status, psychological status, airflow limitation) did not differ in patients, regardless of clinical control status. All-cause mortality was lower in persistently controlled patients (5.5% versus 19.1%, p = 0.001). The hazard ratio for all-cause mortality was 2.274 (95% CI 1.394–3.708; p = 0.001). Regarding pharmacological treatment, triple inhaled therapy was the most common option in persistently uncontrolled patients (72.2%). Patients with persistent disease control more frequently used bronchodilators for monotherapy (53%) at recruitment, although by the end of the follow-up period, 20% had scaled up their treatment, with triple therapy being the most frequent therapeutic pattern.

**Conclusions:**

The evaluation of COPD control status provides relevant prognostic information on survival. There is important variability in clinical control status and only a small proportion of the patients had persistently good control. Changes in the treatment pattern may be relevant in the longitudinal pattern of COPD clinical control. Further studies in other populations should validate our results.

*Trial registration:* Clinical Trials.gov: identifier NCT01122758.

## Background

Over the last decade, we have seen new evidence that has led to a new vision of chronic obstructive pulmonary disease (COPD) with the recognition of the multidimensional component and the concept of phenotype, which has meant a step forward on the road to personalized medicine and individualization of treatment [1–3].

Clinical practice guidelines in COPD establish the reduction of symptoms and minimization of risk as the main therapeutic objectives [4, 5]. These objectives make it necessary to adapt actions to the changes experienced by patients throughout their evolution, considering therapeutic success to mean achieving disease control.

The concept of COPD control is a new dimension that is proposed as a tool to help make therapeutic decisions and to modulate treatment [6, 7]. According to this proposal, control is defined as a state of low clinical impact and an absence of exacerbations maintained over time. The prespecified criteria for clinical control were described by Soler Cataluña [6] and have subsequently been evaluated in several studies [8–10].

Control in COPD is a dynamic concept that can reflect changes in patients’ clinical status that may have prognostic implications. Some studies have observed a potential predictive value for poor outcomes and previous studies have shown that improvement in control status in the short term was associated with better outcomes, improvement in health status, less frequent exacerbations [11] and a longer delay until hospitalization [8]. However, this new concept requires validation in terms of its ability to predict outcomes and to provide additional clinical management insight. Given the limited information about the changes in clinical control in patients with COPD and the relationship with outcomes in those patients, we assessed clinical control at baseline and longitudinally (annually over 5 years) in patients participating in the CHAIN (COPD History Assessment in Spain) cohort, aiming to use CHAIN data to explore the changes and consequences of clinical control in a large cohort of patients with COPD.

We hypothesized that worse persistent control would relate to worse clinical outcomes. We followed longitudinal changes in physiological outcomes and patient-reported outcomes for health status, dyspnea and psychological status over 5 years in patients with COPD. The objectives of the present study were as follows: (1) to evaluate the degree of control in patients with COPD; (2) to provide information on the longitudinal evolution of clinical control and to determine the factors associated with worse control; (3) to validate the concept of control as a predictor of the risk of poor outcomes.

## Methods

The CHAIN methodology has been extensively reported previously [12]. Briefly, CHAIN is a Spanish multicenter study carried out at pulmonary clinics. The main goal of this prospective observational study was to multidimensionally evaluate the progression of patients with COPD to better define the natural history and phenotypes of the disease. The recruitment period began on January 15, 2010, and is ongoing (Clinical Trials.gov: identifier NCT01122758). All participants signed the informed consent approved by the ethics committees of the participating centers (Hospital Universitario la Candelaria, Tenerife; Spain; IRB No. 258/2009). COPD was defined as a smoking history of at least 10 pack-years and an FEV1/FVC ratio less than 0.70 after inhaling 400 mg of albuterol. Patients were stable for at least 6 weeks and received optimal medical therapy. Exclusion criteria were uncontrolled comorbidities such as malignancy or other confounding diseases that could interfere with the study. The follow-up of the subjects included annual office visits and a telephone call was scheduled every 6 months to compile data about the number of exacerbations, clinical impact (health-related quality of life, subjective perception) and to verify the subject’s vital status. COPD treatment followed national [5] and international guidelines. Data analyzed in the present study was obtained from the recruitment date through September 2018. Data was anonymized with hierarchical access control in order to guarantee that information was secure.

### Clinical and physiological measurements

Trained staff obtained information on age, sex, body mass index (BMI) and smoking status at baseline and subsequent visits. Comorbidities were scored using the Charlson index [13]. Pulmonary function tests were performed according to international criteria [14, 15]. Dyspnea was evaluated using the modified Medical Research Council (mMRC) scale [16]. To evaluate health-related quality of life, the Spanish validated version of the COPD Assessment Test was used, which was self-administered by each patient under the supervision of the interviewer [17]. Anxiety and depression were evaluated using the Hospital Anxiety and Depression Scale (HAD) questionnaire [18]. Exacerbations were defined as a worsening of respiratory symptoms (dyspnea, cough or sputum) that required the use of antibiotics, systemic corticosteroids, or both, or symptoms that necessitated an emergency room visit or hospital admission. All-cause mortality was recorded using information obtained from the family and then confirmed by reviewing the medical record.

### Clinical control status assessment

Control status was evaluated based on low clinical impact and stability, according to clinical criteria. A patient was considered controlled when disease was clinically stable and had low clinical impact, adjusted for the level of disease severity. Stability was defined as the absence of exacerbations in the previous 6 months plus no change or improvement in subjective perception referred to by the patient. Clinical impact was classified as low according to the information collected on the dyspnea (mMRC) scale (0–1 if FEV1 ≥ 50% and 0–2 if FEV1 < 50%) and rescue medication usage (not needing to use rescue inhalers regularly). The level of control was evaluated longitudinally during visits every 6 months. All participants had a minimum of 12 months of follow-up with clinical control measurements. Based on the clinical control status evaluated at each visit during follow-up, the cohort was divided into three subgroups: persistently controlled, intermittently controlled and persistently uncontrolled patients.

### Statistical analysis

Data is summarized as frequencies for categorical variables, median (5th–95th percentile) for ordinal or non-normal scale variables and mean ± SD for normally distributed scale variables. Comparisons were made between groups using Pearson’s chi-squared test, the Kruskal–Wallis H test or the Mann–Whitney U test and one-way ANOVA or the t-test as appropriate.

Logistic regression was used to investigate factors contributing to clinical control in patients with COPD. A multivariate analysis considered variables with a statistically significant association (p < 0.05). In the multivariate model, we considered the following independent variables: age, pack-years, chronic bronchitis, dark sputum, eosinophils, Charlson index, FEV1, KCO, triple therapy, CAT score and HDAS depression.

We chose the best predictive model, which only had the variables CAT score and triple therapy because the others weren’t as relevant to provide a good model. To select the model, we used the Akaike and Bayesian information criteria. The final set of variables was selected using a backward stepwise selection algorithm (p < 0.10 to remain in the model). The discrimination capacity of the predictive model was analyzed by calculating the area under the Receiver Operating Characteristics (ROC) curve along with a confidence interval at 95%.

An unpaired t -test was used to compare baseline data and annual changes between persistently controlled and persistently uncontrolled status. P values less than 0.05 were considered to be statistically significant.

A Kaplan–Meier analysis for survival due to all causes was performed in persistently uncontrolled patients. Finally, to predict the risk of death, we performed Cox proportional hazard regression analyses with the persistently controlled and uncontrolled subgroups. Significance was established as two-tailed p < 0.05.

## Results

### Participant characteristics

The population of this study was 798 patients with COPD from the CHAIN study who underwent a minimum of 12 months of follow-up with clinical control measurements. Stability was defined as the absence of exacerbations in the last 12 months during the recruitment visit. A total of 264 (33%) patients met the criteria for controlled status at recruitment. A comparison of controlled versus uncontrolled patient characteristics is presented in Table 1. Uncontrolled patients were older and had a higher body mass index and greater degree of airflow limitation, with more chronic bronchitis and the presence of dark sputum, more comorbidities and a poor quality of life. Regarding pharmacological treatment, uncontrolled patients more frequently used inhaled triple therapy.

**Table 1:** Characteristics of the study population according to control status at recruitment

	Total n = 798	Controlled n = 264 (33%)	Uncontrolled n = 534 (66.8%)	P-value
Age (years), m (SD)	65.7 (10.5)	62.8 (11.6)	67.2 (9.7)	< 0.001
Gender (male), n (%)	663 (82.9)	226 (85.6)	435 (81.5)	0.144
Active smoker, n (%)	229 (28.6)	92 (34.8)	136 (25.5)	0.006
Tobacco exposure, pack-years, m (SD)	56.3 (28.7)	52.6 (25.8)	58.2 (29.9)	0.018
BMI (kg/m^2^), m (SD)	28.0 (5.1)	27.2 (4.7)	28.4 (5.2)	0.001
Post-bronchodilator FEV1 (%), m (SD)	60.2 (25.9)	68.0 (20.8)	56.4 (27.3)	< 0.001
Post-bronchodilator FEV1 (mL), m (SD)	1629.9 (690.4)	1944.0 (746.4)	1476.6 (602.7)	< 0.001
K_CO_%, median (P25-P75)	73 (51–92.9)	76.05 (60–95)	70 (46–91)	0.002
Chronic bronchitis, n (%)	466 (58.2)	132 (50.0)	334 (62.5)	0.001
Dark sputum, n (%)	122 (15.2)	29 (11.0)	93 (17.4)	0.018
Bronchial asthma, n (%)	26 (3.3)	11 (4.2)	15 (2.8)	0.309
Eosinophils (%), median (P25-P75)	2.3 (1.5–3.6)	2.4 (1.6–3.6)	2.3 (1.5–3.6)	0.666
Charlson index, m (SD)	1.2 (1.5)	1.0 (1.5)	1.3 (1.5)	0.009
Treatment, n (%)				
Inhaled triple therapy	454 (56.8)	105 (39.8)	348 (65.2)	< 0.001
Theophylline	73 (9.1)	8 (3.0)	65 (12.2)	< 0.001
Influenza vaccine	430 (53.7)	102 (38.6)	328 (61.4)	< 0.001
LTOT	104 (13.0)	9 (3.4)	95 (17.8)	< 0.001
Home ventilation	41 (5.1)	9 (3.4)	32 (6.0)	0.120
CAT score, m (SD)	12.6 (7.2)	10.3 (6.5)	13.8 (7.3)	< 0.001
Anxiety, HDAS, m (SD)	11.1 (4.8)	11.06 (4.8)	11.19 (4.9)	0.576
Depression, HDAS, m (SD)	8.6 (4.7)	8.2 (4.5)	8.9 (4.7)	0.088

*BMI* body mass index, *FEV1* forced expiratory volume in 1 s, *K*_*CO*_ carbon monoxide transfer coefficient, Inhaled triple therapy: long-acting beta-2 agonist (LAMA) with corticosteroids (ICS) with long-acting antimuscarinic agent (LAMA), *LTOT* long-term oxygen therapy, *CAT* COPD Assessment Test, *HDAS* Hospital Anxiety and Depression Scale

#### Control status according to degree of airflow limitation at recruitment

Of a total of 300 patients with severe/very severe airflow limitation, 228 patients (76%) were defined as having low-impact disease and 100 patients (33.3%) had stable disease; therefore, 26.7% were defined as controlled patients. In mild/moderate COPD, there was a greater proportion of patients with stable disease: 262 patients (52.6%). Of these, 36.9% patients were defined as controlled (Table 2).

**Table 2:** Factors accounting for the control status of patients with COPD by level of severity at recruitment

	Total (n = 798)	FEV1 ≥ 50% (n = 498)	FEV1 < 50% (n = 300)	P-value
Clinical impact				< 0.001
Low, n (%)	546 (68.4)	318 (63.9)	228 (76.0)	
High, n (%)	252 (31.6)	180 (36.1)	72 (24.0)	
Stability				
Stable, n (%)	362 (45.4)	262 (52.6)	100 (33.3)	
Not stable, n (%)	436 (54.6)	236 (47.4)	200 (66.7)	< 0.001
Control status				
Controlled, n (%)	264 (33.0)	184 (36.9)	80 (26.7)	
Uncontrolled, n (%)	534 (67.0)	314 (63.1)	220 (73.3)	0.003

*FEV1* forced expiratory volume in 1 s

#### Prevalence and longitudinal follow-up of clinical control

**Fig. 1:**
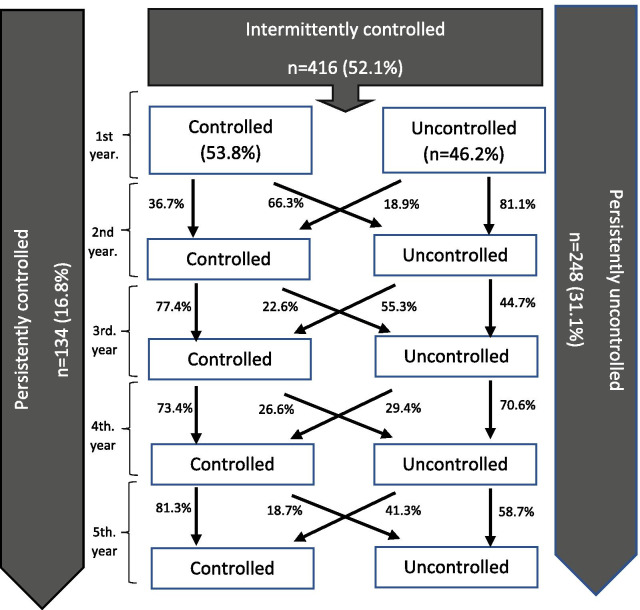
The evolution of the clinical control pattern every year

Over a period of 5 years, the proportion of persistently controlled patients with COPD was 16.8%, persistently uncontrolled patients accounted for 31.1% and intermittently controlled patients represented 52.1% (Fig. 1). There were significant differences in baseline clinical and physiological characteristics between the persistently controlled patients with COPD compared to those who were persistently uncontrolled or intermittently controlled (Table 3).

**Table 3:** Baseline characteristics of longitudinal clinical control patterns

	Persistently controlled (n = 134)	Intermittently controlled (n = 416)	Persistently uncontrolled (n = 248)	P-value
*Demographics and clinical data*				
Male, n (%)	117 (87.3)	336 (80.6)	209 (84.3)	0.153
Age (years), m (SD)	63.2 (9.7)^a,b^	64.9 (10.9)	68.5 (9.9)	< 0.001
Pack-years, m (SD)	53.5 (26.1)	54.6 (28.4)	60.8 (30.2)^c^	0.013
Active smoker, n (%)	48 (35.8)	115 (27.6)	66 (26.6)	0.128
BMI (kg/m^2^), m (SD)	27.1 (4.6)	28.0 (4.8)	28.5 (5.6)	0.060
Chronic bronchitis, n (%)	71 (53.0)^d^	233 (55.9)	162 (65.3)	0.022
Dark sputum, n (%)	15 (11.2)^d^	54 (12.9)	53 (21.4)^c^	0.005
Bronquial asthma, n (%)	5 (3.7)	9 (2.2)	12 (4.8)	0.164
Eosinophils (%), median (P25-P75)	2.6 (1.6–3.9)^d^	2.4 (1.6–3.6)	2.1 (1.3–3.3)^c^	0.002
Charlson index, m (SD)	1.2 (1.6)	1.0 (1.4)	1.5 (1.6)^e^	0.002
*Physiology*				
FEV1 (L), median (P25-P75)	2050 (1467–2505)^b^	1600 (1190–2050)^a^	1360 (940–1730)£	< 0.001
FEV1%pred, median (P25-P75)	72 (55–88)^b^	60 (46–74)^a^	51 (39–63)£	< 0.001
FVC (L), median (P25-P75)	3585 (2847–4380)^b^	3100 (2450- 3710)^a^	2745 (2197–3227)^e^	< 0.001
FVC %pred, median (P25-P75)	94 (80–110)^b^	84 (71–101)^a^	75 (63–90)^e^	< 0.001
FEV1/FVC, median (P25-P75)	58 (49–65)^b^	54 (44–63)^f^	51 (41–60)^c^	< 0.001
K_CO_%, median (P25-P75)	79.5 (62.5–99.7)^b^	72.6 (52.2–92.8)^f^	66 (41–85.2)^c^	< 0.001
*Treatment*				
Triple therapy, n (%)	50 (37.3)^b^	226 (54.2)^f^	178 (71.8)^e^	< 0.001
Influenza vaccine, n (%)	50 (37.3)^b^	213 (51.1)^f^	167 (67.3)^c^	< 0.001
LTOT, n (%)	3 (2.2)^b^	37 (8.9)	64 (25.8)^e^	< 0.001
VMNI, n (%)	5 (3.7)^b^	12 (2.9)	24 (9.7)^c^	< 0.001
CAT score, median (P25-P75)	8 (5–14.2)^b^^,f^	11 (7–16)	14 (9–21)^e^	< 0.001
HDAS anxiety score, median (P25-P75)	11 (6–15)	12 (8–15)	12 (8–15)	0.576
HDAS depression score, median (P25-P75)	8.0 (4.6)	8.5 (4.5)	9.3 (4.9)	0.048
Follow-up time (years), m (SD)	2.4 (1.7) ^d^	4.2 (1.2)^a^	1.8 (1.3)^e^	< 0.001

*BMI* body mass index, *FEV1* forced expiratory volume in 1 s, *FVC* forced vital capacity, *K*_*CO*_ carbon monoxide transfer coefficient, *Triple therapy* long-acting beta-2 agonist (LAMA) with corticosteroids (ICS) with long-acting antimuscarinic agent (LAMA), *LTOT* long-term oxygen therapy, *CAT* COPD Assessment Test, *HDAS* Hospital Anxiety and Depression Scale

^a^p < 0.001 persistently controlled compared with intermittently controlled

^b^p < 0.001 persistently controlled compared with persistently uncontrolled

^c^P ≤ 0.05 persistently uncontrolled compared with intermittently controlled

^d^P < 0.05 persistently controlled compared with persistently uncontrolled

^e^P ≤ 0.001 persistently uncontrolled compared with intermittently controlled

^f^p ≤ 0.05 persistently controlled compared with intermittently controlled

During this follow-up over 5 years, the median follow-up time in the persistently controlled patient group was 2.4 (1.7) years, 4.2 (1.2) years in the intermittently controlled group and 1.8 (1.3) years for persistently uncontrolled patients. The loss of patients during follow-up was 35.7%.

#### Factors accounting for persistently controlled patient status

A backward logistic multivariate model was developed with persistent control as the independent variable and the dependent variables were clinical and demographic variables, which were not related to the definition of control. The adjusted model showed that triple therapy (OR, 0.3026; 95% CI, 0.1776–0.51573; p < 0.001) and CAT (OR, 0.9399; 95% CI 0.9032–0.9781; p < 0.001) were independently and significantly associated with persistently controlled status. The AUC was 0.7029 (95% CI, 0.64209–0.76367).

#### Changes in treatment patterns for COPD in persistently controlled and uncontrolled patients

**Fig. 2:**
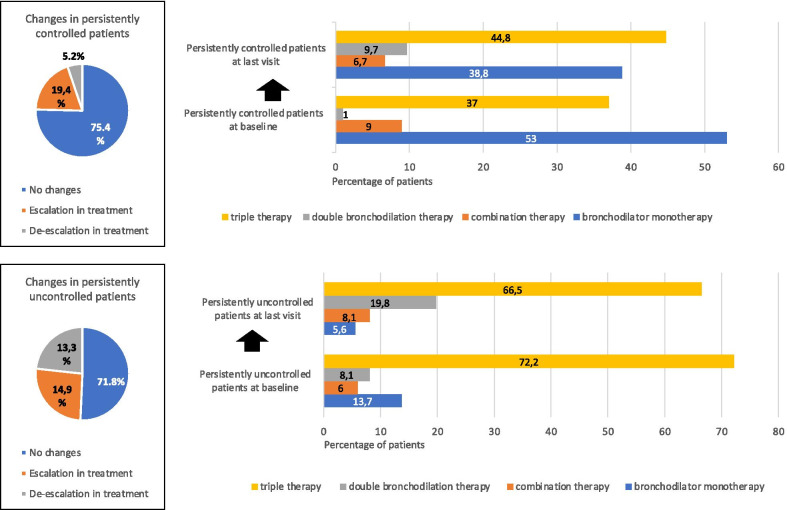
Changes in treatment patterns for COPD at baseline and last visit in persistently controlled and uncontrolled patients

Regarding pharmacological treatment, persistently uncontrolled patients more frequently used inhaled corticosteroids, particularly as part of triple therapy (72.2%). Of these, 71.8% showed no changes in treatment during follow-up, 13.3% underwent de-escalation and 14.9% escalation in treatment. Patients who were persistently controlled more frequently used bronchodilators, particularly monotherapy (53%), followed by triple therapy (37%). Of these, 5.2% de-escalated treatment and 19.4% scaled up their treatment, with triple therapy being the most frequent therapeutic pattern (Fig. 2).

#### Outcomes in patients with COPD according to longitudinal control status pattern

Longitudinal changes in clinical outcomes (health status, psychological status and airflow limitation) according to a persistently uncontrolled or controlled longitudinal control status pattern are shown in Table 4.

**Table 4:** Comparisons of baseline data and annual changes between persistently controlled patients and persistently uncontrolled patients during 5 years of follow-up

	Baseline data		Annual changes (/year)	
Characteristics	Persistently controlled	Persistently uncontrolled	Persistently controlled	Persistently uncontrolled
CAT score	9.6 (5.9)	15.5 (7.8)*	0.0 (− 1.0–1.7)	0.2 (− 2.5–3.0)
HDAS anxiety	10.7 (4.8)	11.1 (4.8)	0.0 (− 1.0–2.0)	0.3 (− 1.0–3.0)
HDAS depression	8.0 (4.6)	9.3 (4.9)*	0.5 (− 0.3–2.3)	0.0 (− 1.6–3.0)
FEV1, %pred	71.2 (20.8)	52.3 (19.9)*	0.3 (− 2.8–3.0)	− 0.2 (− 3.4–1.5)

*CAT* COPD Assessment Test; *HDAS* Hospital Anxiety and Depression Scale; *FEV1* forced expiratory volume in 1 s

Data is presented as mean (SD) or median (5th–95th percentile)

*Statistically significant differences between persistently uncontrolled and persistently controlled patients (p < 0.05)

Regarding the baseline data, persistently uncontrolled patients were significantly worse as rated by CAT and HDAS scores and FEV1 levels. However, there were no significant differences in annual changes in outcomes between persistently controlled and uncontrolled patients.

**Fig. 3:**
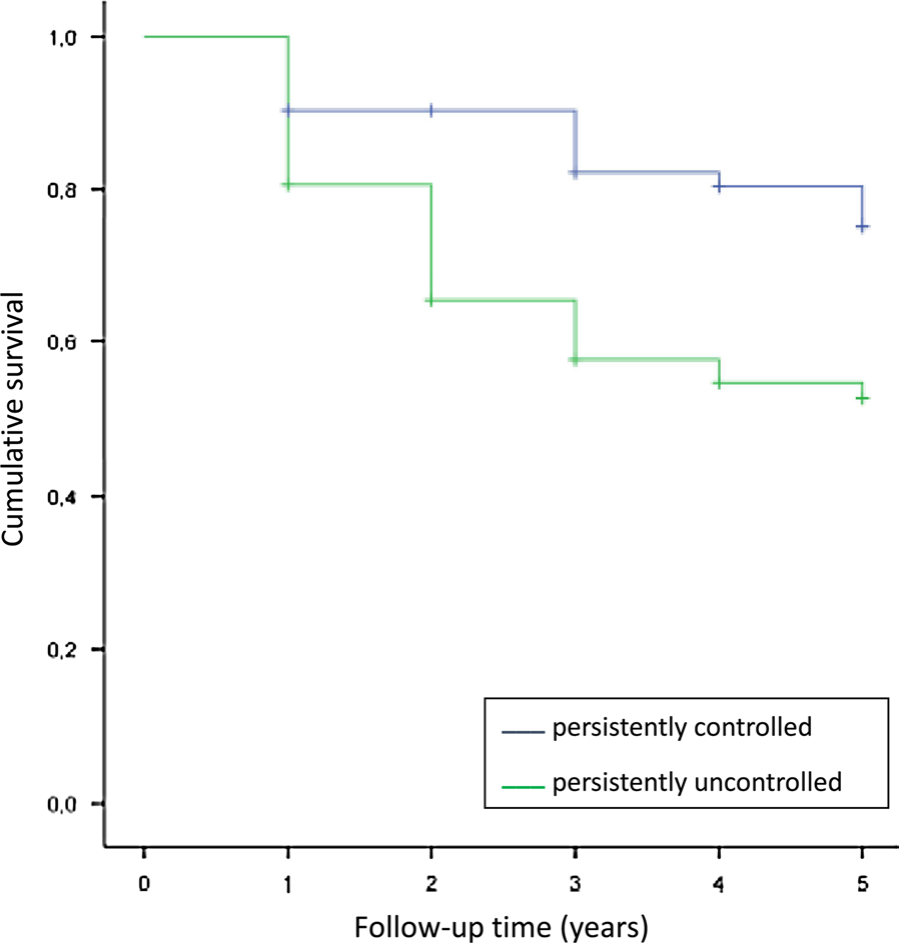
Kaplan–Meier analysis for all-cause mortality. Persistently controlled patients were associated with a longer survival time than persistently uncontrolled patients

Regarding survival, there were 94 (24.6%) deaths in 382 patients with a persistently uncontrolled or controlled status pattern, of which 73 (19.1%) were persistently uncontrolled and 21 (5.5%) were persistently controlled (p = 0.001). The Kaplan–Meier analysis for all-cause mortality showed that persistently uncontrolled status was associated with a shorter survival time (3.58 years; 95% CI, 3.31–3.85) than persistently controlled status (4.43 years; 95% CI, 4.19–4.67) (Fig. 3). The hazard ratio for all-cause mortality was 2.274 (95% CI, 1.394–3.708; p = 0.001).

## Discussion

This study provides novel information on the longitudinal evolution of clinical control in a large cohort of patients with COPD as well as factors associated with persistent clinical control and their clinical consequences.

The main results of our study indicate three things. First, in the population with COPD, there were frequent changes in clinical control status. Only a small percentage of patients could be classified as persistently controlled over the following 5 years. Secondly, the main variables associated with persistent clinical control are a better quality of life as evaluated by the CAT and not requiring inhaled triple therapy. Finally, the clinical consequences of persistent clinical control are observed in the risk of death.

The current analysis describes the progression of clinical control in a well-characterized COPD cohort over a period of 5 years as monitored at pulmonary clinics. In our study, only 33% of patients with different degrees of COPD severity met the criteria required to be considered controlled at recruitment. In the mild or moderate subgroup of patients, 36.9% were defined as controlled whereas only 26.7% of severe patients were defined as controlled. These results are similar to those obtained in an international multicenter study, obtaining an overall control value of 32% using the clinical evaluation of control criteria [10]. Another prospective study showed similar results, with only 27.5% [8] of patients being considered controlled. However, it should be mentioned that unlike these studies, almost 40% of the sample analyzed in our study had severe airflow obstruction. In addition, the level of physical activity referred to by the patient and the presence of sputum purulence were not included in the clinical impact assessment. In our study population, the use of rescue medication as a high impact criterion was present in 70% of patients classified as having a high clinical impact. This is a widely justified criterion if we fear that the increased use of rescue medication has been associated with an increased risk of future exacerbations [19]. However, sputum color has shown the lowest discriminative property for the level of impact [10]. In our study, dark sputum was present in 11.2% of persistently controlled patients compared to 21.4% of persistently uncontrolled patients (p = 0.015).

Regarding the longitudinal clinical control patterns, we found that there were frequent changes in clinical control status, with 42.1% of patients changing control status during the observation period. Few studies have shown data on the progression of clinical control in COPD. A recent international study showed that 53.7% of patients changed control status, 29.8% of patients remained controlled and 16% persistently uncontrolled during an 18-month follow-up [20]. These results are not comparable to our analysis, where follow-up is greater. Another observational study analyzed changes in control over a 3-month period and showed that 29.2% changed their control status [11]. In this study, these changes were significantly more frequent than changes in GOLD stage, risk level or in phenotype, which further suggests that control status could be used as a supplementary assessment tool for decision-making at each medical visit, similar to the evaluation of asthma control. Table 5 summarizes studies that examined the proportion of controlled patients and changes in clinical control.

**Table 5:** Summary of studies that examined the distribution of control status, changes in clinical control and evaluated the predictive value of control

	Number of patients Study design	Investigation objective	Study findings
Baloira A et al. [9] (2016)	481 patients Spanish cross-sectional multicenter study (primary care vs respiratory care)	Distribution of control status	36.8% of patients were controlled
Nibber A [9] et al. (2017)	2788 patients Retrospective observational cohort study	To validate the concept of control Distribution of control status	4.5% of patients were controlled Time to first exacerbation was longer for controlled patients (p < 0.001)
Miravitlles M [10] et al. (2018)	314 patients Multicenter prospective observational study	To validate concept of control Distribution of control status	32% of patients were controlled
Soler-Cataluña JJ [8] et al. (2018)	265 patients Spanish multicenter prospective observational study	To validate “modified” control criteria To evaluate predictive value of control	61.5% of patients were controlled The time to the first combined event (emergency room visit, hospitalization, or death) was significantly greater in controlled patients (p < 0.001)
Barrecheguren [31] et al. (2020)	2044 patients Multicenter double-blind SPARK study	To validate the prospective value of control	20% of patients were controlled The rate of exacerbations was lower in controlled patients (OR 0.56, p < 0.0001) and time to first exacerbation was significantly delayed
Miravitlles [32] et al. (2020)	307 patients International, multicenter study	To validate the concept of control in COPD	65% of patients were controlled Time to first exacerbation was significantly delayed for controlled patients
Soler-Cataluña JJ [11] et al. (2020)	354 patients Prospective multicenter observational study	To compare changes in control over a 3-month period with changes in risk level and GOLD stage	50.3% of patients were controlled Changes in control over a 3-month period was 29.3%
Miravitlles M [20] et al. (2020)	267 patients International multicenter study Follow-up for 18 months	To describe the changes in control status during follow-up (18 months) and the predictive value of control (6 months)	During 18 months of follow-up, 29.8% of patients remained controlled, 16% persistently uncontrolled and the remaining 53.7% changed control status during follow-up

In our study, 31.1% of patients had persistently poor disease control during follow-up and only a small proportion (16.8%) of patients had persistently good control. We found that persistently controlled patients were younger, had less frequent chronic bronchitis, a lower degree of airflow obstruction, lower involvement in the diffusion test, a better quality of life as evaluated by the CAT and a higher level of peripheral eosinophilia. In previous studies [9, 10, 20, 21], the presence of chronic bronchitis, female sex, lower BMI and a history of prior exacerbations were identified as variables that were significantly associated with poor control. In addition, poor lung function and worse health status were demonstrated to be the best predictors of the risk of future exacerbations and were associated with a significant increase in the risk of mortality [22]. However, our study found that sex, tobacco history, BMI and comorbidities such as bronchial asthma or anxiety and depression were similar in patients, irrespective of longitudinal clinical control status. These results are similar to those reported by Calverley et al. [23], who showed that tobacco history and BMI were similar in individuals with frequent exacerbations and those who never experienced an exacerbation over the 2 years of follow-up. However, continued smoking in patients with COPD has been associated with higher disease impact and increased exacerbations [24]. In addition, former smokers had a significantly reduced risk of death and hospitalization compared to active smokers [25]. In our study, the majority of the patients maintained their tobacco use status. There were no differences in longitudinal clinical control patterns regarding smoking cessation during follow-up.

The use of maintenance respiratory therapy is usually thought to reduce risk. However, data reported in the ECLIPSE [26] and SPIROMICS [27] cohorts reported that patients did shift from high-risk to low-risk groups over time, though the reasons for doing so were unclear. In any case, adequate therapy seems to improve the ratio of infrequent to frequent exacerbators over time [28–30]. In our study, triple therapy at baseline was less frequent in persistently controlled patients (37%) versus persistently uncontrolled patients (72.2%). At the end of the follow-up period, 20% of persistently controlled patients had scaled up their treatment, with triple therapy being the most frequent therapeutic pattern. On the contrary, in persistently uncontrolled patients, 13.3% had increased their pharmacological treatment while 15% had decreased it, observing a decrease in triple therapy and an increase in double bronchodilator therapy. These results for the changes in treatment pattern according to longitudinal control status provide interesting information, showing an increase in triple therapy in persistently controlled patients. In our study, not requiring triple therapy at baseline and having a better quality of life were identified as variables that were significantly associated with persistent disease control. A likely explanation why patients are given triple therapy to prevent exacerbations is because they are believed to be progressing more poorly and are thus more likely to relapse in the future, irrespective of any positive effect of their therapy.

A previous publication described control status as a marker of increased risk of poor outcomes in the short term. According to data reported in the studies by Soler-Cataluña et al. [8] and Barrecheguren et al. [31], controlled patients showed a lower risk of complications, with a longer delay until the first combined event, the first exacerbation and hospitalization, as well as better health status at 1 year of follow-up. However, they did not report any significant difference in survival between controlled and uncontrolled patients. In the Miravitlles et al. [20] study, uncontrolled patient visits resulted in a highly significant increased risk of poor outcomes over the next 6 months, with an OR of 4.25 for hospitalization due to exacerbation compared to controlled patient visits. In addition, it has been reported that control status determined by clinical criteria was a better predictor of exacerbations compared to CAT criteria (AUC: 0.67 vs 0.57) [32]. Our analysis showed that although a further worsening in CAT and HDAS scores and FEV1 levels was observed in persistently uncontrolled patients, there were no significant differences in annual changes between persistently controlled and uncontrolled subjects. However, we found that persistently controlled patients had a significantly lower risk of death than those who were persistently uncontrolled. In our study, there were 94 (24.6%) deaths in 5 years of follow-up, a mortality rate similar to that of the Spanish PAC-EPOC cohort (3.6 fatal events/year/100 patients) [33]. Specifically, there were 73 deaths in persistently uncontrolled patients and 21 in controlled patients. Our analysis further confirmed that subjects who died were older, had a greater degree of airway obstruction, and had worse health status than those who survived. These results are similar to those reported by Oga et al [34]. Changes in mortality occur after the first year and tend to increase in the second year, which could explain why this was not observed in previous studies [8].

Our study extends our understanding of the concept of control in COPD and its possible application in clinical practice. Previous studies have found that improvement in control status in the short term was associated with better outcomes, with a reduced frequency of exacerbations and improved health status. Our results show that patient control status frequently changes in subsequent clinical visits and we observed that there are long-term consequences: persistently uncontrolled patients have higher mortality. This is the first study to show the impact of control status on long-term mortality. This increased risk justifies the use of control evaluation as a warning sign to foster more careful evaluation of the patients and the adoption of therapeutic measures.

This study has several strengths. It included a large number of well-characterized patients being treated for COPD in “real life” with a long follow-up time, providing invaluable information on outcomes which is not usually available in most pharmacological trials. However, it is necessary to keep in mind some characteristics of the cohort in order to correctly interpret our results. The CHAIN cohort was obtained from an observational study of patients visiting pulmonary clinics and not from general medical practice. In fact, patients with COPD treated in a specialized clinic have been found to have better clinical control [35]. In the Baloira et al. [36] study, patients at the primary care level were more poorly controlled. However, our cohort included a large population of patients with different degrees of severity (16.4% mild, 46% moderate, 26.8% severe and 10.8% very severe). Another consideration is that few women were included in the cohort and the findings reported in relation to this must be interpreted with caution. There was also a loss of patients during follow-up that could result in measurement bias. Regarding the limitations of the present study, it is important to consider that the probability of change in clinical control status will be greater for a longer follow-up period. In our analysis, a minimum of 1 year of follow-up was established as a criterion to define the longitudinal pattern since our objective was to explore the differences between persistently controlled and uncontrolled classifications and to analyze their prognostic implications such as mortality. In this sense, it is worth mentioning that in our analysis, there was a higher number of exitus in the first year of follow-up: 48 patients defined as persistently uncontrolled and 13 as persistently controlled. In addition, if we establish a minimum of 3 years of follow-up as a criterion, the majority of patients (76.8%) would be classified as intermittently controlled. Therefore, we defined the longitudinal pattern with a minimum of 1 year of follow-up, also keeping in mind that this criterion perhaps most closely resembles ordinary clinical practice. Another limitation is that this was not an interventional study, we could not investigate whether a change in treatment could modify control status and influence the outcomes. This has to be demonstrated in future interventional studies.

## Conclusions

This is the first study to show the impact of control status on long-term mortality. There is important variability in clinical control status and only a small proportion of patients had persistently good control. The study highlights the significantly increased risk of death in uncontrolled patients. Consequently, control criteria should be incorporated into clinical practice as a simple tool to help reassess patients with COPD at each follow-up visit. Further studies in other populations should validate our results.

## Data Availability

Data can be shared upon request by writing to mcallerubio@gmail.com.

## Abbreviations

AECOPD: Acute exacerbations of COPD
BMI: Body mass index
CATTM: COPD assessment test
COPD: Chronic obstructive pulmonary disease
ICS: Inhaled corticosteroids
IQR: Interquartile range
LABA: Long-acting beta-2 agonists
LAMA: Long-acting antimuscarinic agents
mHealth apps: Health-related mobile applications
mMRC: Modified Medical Research Council
